# Control of binding of C_60_ molecules to the substrate by Coulomb blockade

**DOI:** 10.1038/s41598-019-52544-4

**Published:** 2019-11-05

**Authors:** Sergey I. Bozhko, Killian Walshe, Igor V. Shvets

**Affiliations:** 1 Institute of Solid State Physics, Russian Academy of Sciences, Chernogolovka, Moscow District, 142432 Russia; 2 School of Physics and Centre for Research on Adaptive Nanostructures and Nanodevices (CRANN), Trinity College Dublin, Dublin 2, Ireland

**Keywords:** Electronic properties and materials, Electronic structure of atoms and molecules, Surfaces, interfaces and thin films

## Abstract

We report on a transition in a monolayer of *C*_60_ molecules deposited on a WO_2_/W(110) substrate. The transition from a static state, where the molecules are rigidly bound to the surface by a coordination bond, to a state where the molecules are loosely bound to the surface by van der Waals force and rotate continuously, has been studied using scanning tunnelling microscopy (STM). The separation between the molecules and the surface increases by 1.2 Å across the transition. The transition from the static state into the rotating state takes place at 259 *K*. The energy of the spinning state with respect to the lowest energy state, having a single coordinated bond, can be obtained from the statistics of the molecules switching. The binding energy of the molecule in the spinning state can be easily altered by changing the polarity of the bias voltage applied between the STM tip and the surface. The binding energy decreases by 80 *meV* when the bias polarity of the sample changes from positive to negative with respect to the tip. The results are consistent with the Coulomb blockade model: when electrons travel from the surface to the *C*_60_ molecule, and then to the tip; charge accumulates on the molecule due to the Coulomb blockade. This increases the electrostatic interaction between the molecule’s charge and a corresponding image charge generated on the metallic surface.

## Introduction

The concept of constructing devices exploiting the electronic properties of single molecules is promising. Organic molecules deposited on insulating substrates in the form of single or fractional (incomplete) monolayers are very attractive systems for making progress in this direction. As the electronic properties of a single organic molecule on a surface are determined by the way it is bound to the substrate, the control of molecule-to-substrate interaction and atomic scale positioning on the substrate is of central importance. Due to the small mass and small moment of inertia, thermal movements of the molecules and thermally activated rotation of the molecules on the surface can hinder the operation of a single molecule device. This raises the importance of understanding the binding, movement and rotation of organic molecules on substrates. We have recently studied a monolayer of *C*_60_ molecules deposited on surface of WO_2_/W(110) using ultra-high vacuum STM^1–3^. The molecules were found in conducting and nonconducting states, and our results indicated that such changes were associated with the molecule acquiring or losing a single electron, i.e. *C*_60_ on the surface could be charge-neutral, or it could gain/lose one electron. This effect can be understood in terms of Coulomb blockade blocking electron transport from the surface to the STM tip via the molecule. We have also established that when such a change in the conduction state of the molecules occurs, the molecule can switch between different orientations at the surface^4^. The change between static and spinning states of the molecules takes place at the temperature of 259 *K*. In a monolayer of *C*_60_, the change takes the form of a phase transition, similar to the one in the *C*_60_ crystal^5–8^, where the molecules of an entire monolayer switch from a static state to a spinning state^2,3^. In this paper, we investigate the nature of the electronic bonds of *C*_60_ molecules positioned on the atomically flat substrate when the molecules undergo the phase transition from the static to the spinning state. We demonstrate that at a certain temperature the molecules become loosely bound to the surface and at once initiate their rotational movement. This change in the binding energy between the molecule and the surface is accompanied by the molecule acquiring a charge and this can be controlled via STM tip bias. Sending electrons from the tip into the *C*_60_ (positive sample bias) activates molecular rotation while reversing the current direction (negative sample bias) suppresses the rotation. The results can be explained on the basis of Coulomb blockade charging of the molecule, leading to a change in the Coulomb interaction force between molecule and substrate.

## Results and Discussion

**Figure 1:**
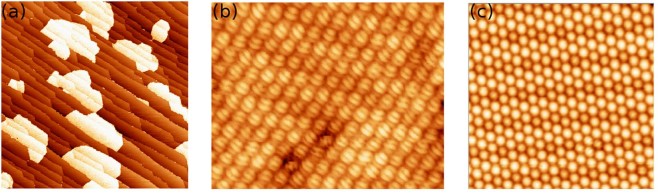
**(a)** 390 × 390 *nm*^2^ Low-temperature STM images acquired after the deposition of 0.5 *ML* of *C*_60_ molecules onto the WO_2_/W(110) surface. *V*_*b*_ = 1 *V*, *I*_*t*_ = 0.1 *nA*. **(b)** 13 × 10 *nm*^2^ Image of *C*_60_ at low temperature with the details of the sub-molecular structure. *V*_*b*_ = 0.97 *V*, *I*_*t*_ = 0.07 *nA*, *T* = 78 *K*. **(c)** 16 × 16 *nm*^2^ STM image of the same *C*_60_ film acquired at *T* = 315 *K*. All molecules in **(c)** appear as perfect spheres due their fast rotation. *V*_*b*_ = −1.4 *V*, *I*_*t*_ = 0.1 *nA*.

A sub-monolayer film of *C*_60_ molecules deposited on the WO_2_/W(110) surface forms one molecule thick nano-islands, (Fig. 1a). Within the nanoislands the molecules are self-assembled into a close-packed hexagonal structure^1^ with 1 nm separation between *C*_60_ molecules, Fig. 1b.

**Figure 2:**
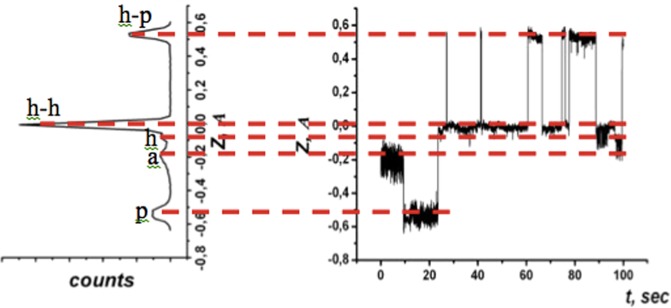
A tunneling microscope tip is located above a fluctuating *C*_60_ molecule. Right panel: The time-evolution of the STM tip-surface distance for the switching *C*_60_ molecule measured at a sample bias, *V*_*b*_ = −1.1 *V* and tunneling current *I*_*t*_ = 0.087 *nA*. The acquisition time was 10 *ms* per point. Five different states are present indicated by dashed lines. Left panel: Histogram of *C*_60_ molecule residence in different states.

At temperatures below 220 *K*, the molecules are completely static on the time scale of the STM experiment thus making it possible to reveal their sub-molecular electron orbital structure. The sub-molecular structure changes from one molecule to another as the molecules can be attached to the surface by 1, 2, 5 or 6 carbon atoms, known by rotational orientations ***a***, ***h-p***, ***h-h***, ***p*** and ***h***. Most molecules are found in the orientations corresponding to ***h-h*** attachment, see Fig. 1b. The specific attachment can be identified on the basis of comparison between density functional theory (DFT) calculations and the STM images^2^. Once the temperature is raised to 220 *K*, the molecule becomes unstable in any one binding position as the thermal energy of the molecule becomes comparable with the barrier separating the positions. Between temperatures of 220–260 *K*, the transitions between different molecular binding positions occur rarely enough so that the individual orientations can be time-resolved with STM and yet often enough so that changes could be observed during the time of the STM experiment. The transitions are best observed by positioning the STM tip above the molecule with either an open or closed tunnel current feedback loop. Figure 2 shows a molecule switching between five different states corresponding to five rational orientations of the molecule at the surface.

**Figure 3:**
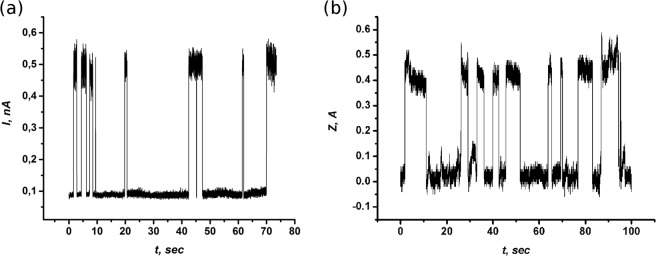
The time evolution of the STM current **(a)** and tip-surface distance **(b)** for the switching *C*_60_ molecule between two lowest in energy orientations when the tunneling microscope tip is located above a fluctuating *C*_60_ molecule. *V*_*b*_ = −1.1 *V*.

**Figure 4:**
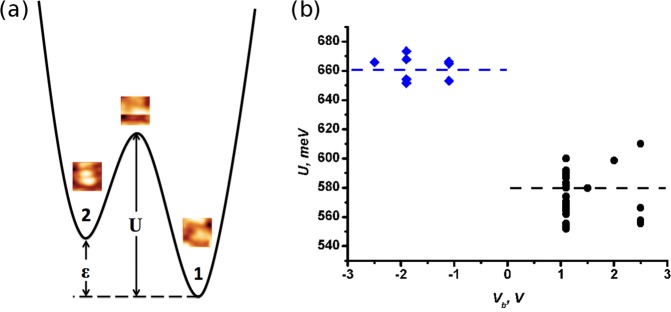
(**a**) Model for two binding states of a *C*_60_ molecule. STM images of the *C*_60_ molecule before, during and after the switching are presented in inserts. **(b)** Dependence of potential barrier separating two adjacent in energy *C*_60_ molecule’s orientations on the applied bias voltage.

The switching of the molecule between 5 rational orientations is detected as vertical height (z-position) change of the STM tip which can be seen in the right panel of Fig. 2. The current feedback remains on in this case. The transitions are manifested as changes in the z-position of the probe due to change of the *C*_60_ molecule’s orientation. The state where the molecule spends the longest time on average is the state with the lowest binding energy of the molecule. With reference to Fig. 2, this is concluded to be the ***h-h*** state. The majority of switching events observed in the experiment were due to the change of molecule orientation between the two lowest energy binding states. In Fig. 3a the STM probe is positioned directly above one molecule, the bias voltage is fixed at −1.1 *V* and the current feedback is disabled so that the tip height remains constant. The change in the tunnelling current due to the *C*_60_ molecule switching is observed in Fig. 3a. Such a mode of observation provides a faster response time of the STM. However, one could also operate the instrument with the feedback switched on and measure the z-position of the tip, see Fig. 3b. In any case, it is important that we now observe a transition between the ground state, with the molecule being fixed in one position, and the lowest energy excited state. The change in state of the molecule results in a change of current flowing through it. We shall discuss the details of this and the related mechanism later, for now we focus on the information that could be extracted from the mean time spent by the molecule in each state. Switching between two binding states of *C*_60_, which are adjacent in energy, is well described in terms of a two-valley model^3^. In this model, we consider two potential wells separated by an energy barrier *U* to estimate the molecule’s lifetime in two states, a depiction of this model is shown in Fig. 4a.

The average lifetimes *τ*_1_ (*τ*_2_) of a molecule transitioning from state 1 (ground state) to state 2 and vice versa over a potential barrier separating the two states is determined by the number of attempts to overcome the potential barrier, i.e. Libron frequency *f*_*L*_:

1 $$\frac{1}{{\tau }_{1}}={f}_{L}\cdot {e}^{-\frac{U}{{k}_{b}T}}$$

2 $$\frac{1}{{\tau }_{2}}={f}_{L}\cdot {e}^{-\frac{U-\varepsilon }{{k}_{b}T}}$$

Here, *U* and *U* − *ε* are the heights of the potential barrier seen from the states 1 and 2 respectively. The difference in energies of the two states *ε* can be estimated from a statistical distribution of the residence time of the molecule in each state^3^. The difference in energies was estimated to be 18 ± 9 *meV* and does not depend on the bias which was used in the experiment. What is remarkable is that both potential barriers *U* and *U* − *ε* depend on the polarity of the bias. For positive bias, whereby electrons move from the tip to the molecule and then to the substrate, the energy of the potential barriers are some 80 ± 17 *meV* higher than in the opposing situation: negative bias, with electrons moving from the surface to the molecule and then to the tip. It is also notable that the energy of the molecule in the spinning state is not sensitive to the value of the bias voltage but rather to its polarity. The results shown in Fig. 4b represent statistics based on data collected from 35 molecules. Some scattering of the resulting values for the barrier in the plot could be due to *C*_60_ molecules being located at a number of non-equivalent sites on the WO_2_ surface.

**Figure 5:**
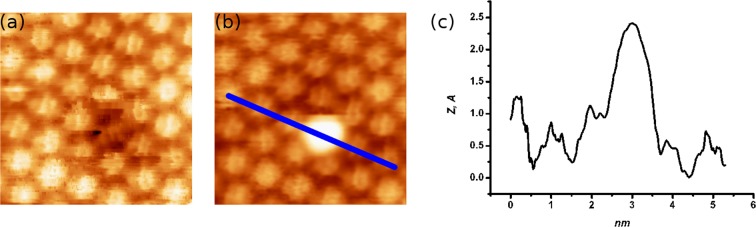
**(a,b)** Constant-current STM images (5.5 × 5.5 *nm*^2^) of the same area of the *C*_60_ monolayer on the WO_2_/W(110) surface, *V*_*b*_ = 1.0 *V*, *I*_*t*_ = 0.1 *nA*. The molecule at the center of these images switches between static and rotating states, changing its appearance. The molecule in **(b)** rotates faster than the time scale of the STM experiment. **(c)** A line profile (along the line marked in **(b)**) indicating the height difference between the rotating and static *C*_60_ molecules in the monolayer.

At high temperatures, above the temperature of the rotational phase transition (*T*_*C*_ = 259 *K*) the molecules rotate faster than the time scale of the STM experiment^2^. The sub-molecular structure can no longer be resolved and the molecules appear in STM as perfect spheres Fig. 1c. If the temperature is somewhat reduced, to around 256 *K*, we can establish a situation where fluctuations appear^2,3^ (see Supplementary video) and the *C*_60_ molecule typically switches between two states: the molecule being fixed on the surface, Fig. 5a and the same molecule rotating on the surface, Fig. 5b. A line profile indicated the height difference between the molecule and the substrate in this rotating state is presented in Fig. 5c.

**Figure 6:**
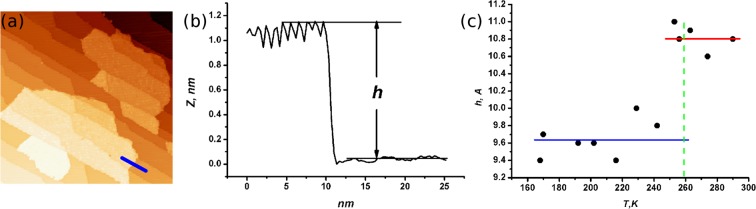
**(a)** 160 × 160 *nm*^2^ STM image of nano-islands acquired after the deposition of 0.5 *ML* of *C*_60_ molecules onto the WO_2_/W(110) surface. *V*_*b*_ = 1.0 *V*, *I*_*t*_ = 0.1 *nA*. **(b)** A line profile (along the line marked in **(a)**) indicating the height of *C*_60_ nano-island. **(c)** Dependence of *C*_60_ nano-island height on the temperature. Abrupt increase of the height at temperature of rotation phase transition indicates break of *C*_60_ coordination bonds.

In the first state, the molecule is static showing the sub-molecular features. This is the ground state as can be judged from the fact the molecule spends most of its time in this state. In the second state, the molecule spins faster than the time resolving capability of the STM. It, therefore, appears as a sphere in the STM image and the overall current through the molecule increases. The most surprising result was obtained when we measured the separation of the *C*_60_ molecules from the surface at different temperatures. The clearest way of doing this was to measure the height of the monolayer thick island of *C*_60_, seen in Fig. 6a by inspecting the step height from the substrate at the edge of the island. A sample line profile is shown in Fig. 6b. The results, presented in Fig. 6c, reveal an abrupt increase of the nano-island height at the temperature of the rotational phase transition (259 *K*) (indicated by the green line). Therefore, as the molecules start spinning at the surface they simultaneously move up and away from the surface and the bond between the molecule and the surface is altered. In the monolayer of *C*_60_ this takes the form of a well-defined phase transition at 259 *K*.

The observed increase in the separation between the molecules and the surface of 1.2 Å is significant. It is in agreement with the difference in rotating and static molecule heights visible in the STM images and comparable with the difference in length between a coordinated and a van der Waals bond (0.9–1.2 Å)^9–11^. This value is much greater than the difference between the bond lengths for the five well-defined static positions of the molecule. The bond lengths obtained from the DFT calculations^2^ are presented in the table presented in (Table 1). The z-position for the ground state of the molecule (***h-h*** orientation) is set as a reference point and the bonds of the other four orientations are given with respect to the ***h-h*** ground state position. The difference in the *C*_60_-WO_2_/W(110) gap for the five orientations is of the order of 0.5 Å or less, significantly smaller than the difference in the separation between the molecules and the surface across the rotational transition at 259 *K*.

**Table 1:** z-position (height above surface) of a *C*_60_ molecule’s center of mass for five rotational orientations with the ground state of the molecule (***h-h*** orientation) set as a reference point.

Orientation	a	h-p	h-h	p	h
Δz, Å	0.23	0.46	0	0.56	−0.15

**Figure 7:**
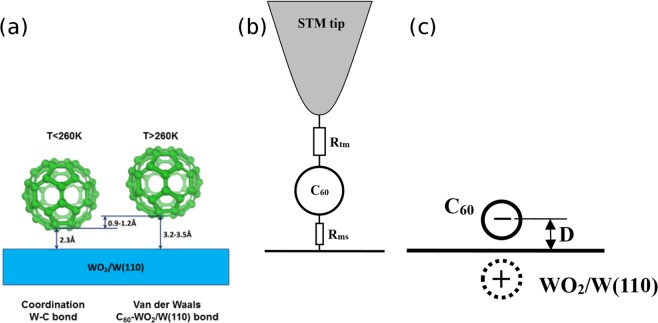
(**a**) Schematics of *C*_60_ molecule bonded to WO_2_/W(110) surface by coordination (on the left) and van der Waals (on the right) forces. (**b**) Equivalent circuit of *C*_60_ molecule coupled in STM tunnelling junction. (**c**) Schematics of image charge of negatively charged *C*_60_.

We have to conclude that during the rotation of the *C*_60_ molecule, the coordinated bond breaks and the molecule remains bonded to the surface via much weaker van der Waals forces, a schematic of this is presented in Fig. 7a. With this in mind, we can explain all the results presented here, including the abrupt dependence of the energy of the rotating state on the polarity of the bias voltage. The model of a molecule bonded to the surface by a weak van der Waals force is shown in Fig. 7b.

The model is characterised by two impedance values^12^: the impedance from the molecule to the surface (*R*_*ms*_) and the impedance from tip to the molecule (*R*_*tm*_). The resistance *R*_*tm*_ has to be much greater than the value *R*_*ms*_, for the STM to operate in a stable regime^13^. In this case one could achieve the conditions for the Coulomb blockade if the electrons flow from the sample to the tip (negative bias) but not in the opposite regime: electrons flow from the tip to the sample (positive bias). Under the conditions of the Coulomb blockade, the *C*_60_ molecule can acquire and retain an extra electron. Once the bias is reversed, the molecule remains uncharged at any tunnelling current value. The charged molecule will create an image charge in the metal, seen in Fig. 7c, which results in a reduction of energy of the charged molecule by $U=\frac{{e}^{2}}{2D}$, where *D* is the distance between the surface and the created charge. *D* can be estimated from DFT simulations (distance between molecule center and W atoms layer) and was taken to be about 8.7 Å. This results in an energy reduction of 414 *meV*. When the coordinated bond breaks and the molecule starts spinning, the change in *D* is Δ*D* = 1.2 Å. This results in a change in the energy barrier of Δ*U* = 50 *mV*, which is close to the experimental value of 80 *meV*. This deviation can be explained by 3 factors: (i) inhomogeneous charge distribution in a frame of the molecule^4,14^; (ii) there is some uncertainty in reference point of *D*; (iii) the electric field between the STM tip and the metal surface also contributes to the potential barrier. Therefore the results suggest that rotation state of the *C*_60_ molecule, which is realized at temperatures above that of the rotational phase transition, is different to any of the fixed binding states, as the energy of the spinning state is controlled by the bias of the tip and the direction of the current flowing through the molecule. The voltage controlling the switch between the static and the rotational states is around 1 *V*, the range convenient for typical electronic devices. The electronic impedance through the molecule is affected by the state of the molecule. This opens up the prospect of using such molecules on a surface as the core of an electronic current control device.

**Figure 8:**
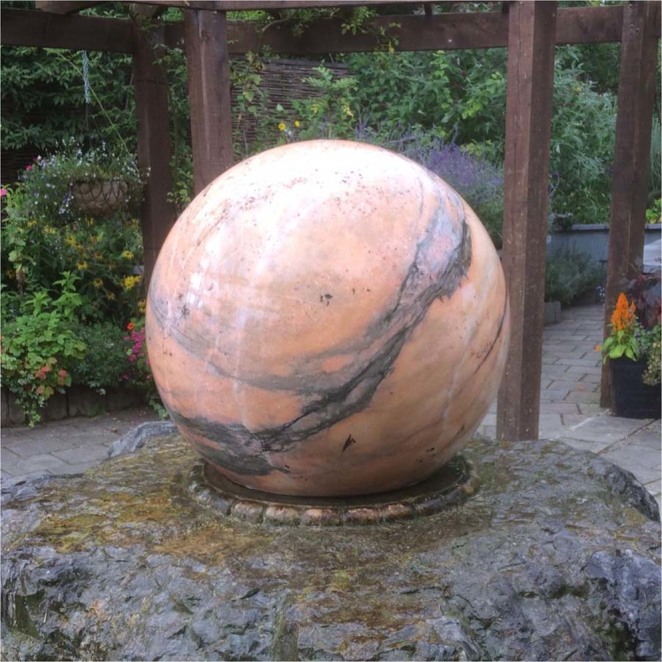
Kugel fountain as a model of rotating *C*_60_ molecule. Image courtesy of M. Grassick.

One could consider the analogy with a Kugel fountain, see Fig. 8. Once the granite sphere is lifted from the surface, it is easily rotated. When it comes down, it is positioned on the surface in a single well-defined position. This is broadly similar to the phase transition we observe for the *C*_60_ molecules on the surface. Once the coordinated bond breaks due to e.g. increased temperature, the molecule lifts away from the surface and it is then retained above the surface by a much weaker van der Waals force. In such a state the molecule is free to spin. The lifting of the molecule can be promoted or demoted by altering its charge. If the molecule is charged, it induces an image charge on the surface and this increases the energy binding the molecule to the surface and thus suppressed the transition from the bound state of the molecule to the spinning state. The charge of the molecule can activate the Coulomb blockade that is controlled by the direction of the current (into the surface or into the tip) and therefore controlled by the polarity of the bias voltage. Using the analogy of the Kugel fountain, this is similar to regulating the water pressure underneath the granite sphere: if the pressure is reduced, the sphere’s ability to go into the rotating state is suppressed. The observed difference in the potential barrier separating two adjacent in energy *C*_60_ molecule orientations, when the applied bias voltage changes polarity opens a way to control the rotation of the *C*_60_ molecules.

**Figure 9:**
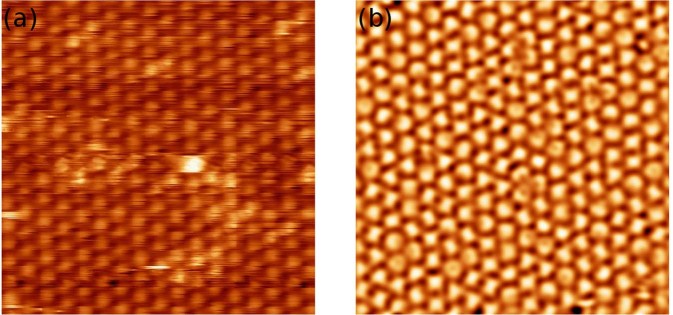
Constant-current STM images (14 × 14 *nm*^2^) of the same area of the *C*_60_ monolayer on the WO_2_/W(110) surface, *I*_*t*_ = 0.1 *nA* **(a)** *V*_*b*_ = 1.2 *V*; **(b)** *V*_*b*_ = −1.9 *V*.

An STM image, Fig. 9a, acquired close to the rotational phase transition point at positive bias voltage (*V*_*b*_ = 1.2 *V*) is noisy and does not reveal the internal structure of molecules due to frequent switching of the molecules or their rotation. However, an STM image, presented in Fig. 9b, of the same area at *V*_*b*_ = −1.9 *V* demonstrates prominent internal structure of *C*_60_ molecules, which corresponds to molecular symmetry.

## Conclusion

We have studied the transition of *C*_60_ molecules deposited on a WO_2_/W(110) substrate from a fixed state, where the molecules are bound to the surface by a well-defined bond, to another state, where the molecules are loosely bound to the surface by van der Waals forces. The integrity of the monolayer of *C*_60_ molecules is not altered once the molecules transition to the loosely bound spinning state, but remarkably, the separation between the molecules and surface is increased by 1.2 Å across the transition. In the loosely bound state, the molecules start spinning around faster than the time resolving capability of the STM and therefore they appear in the STM as perfect spheres. In contrast, at low temperature, the molecules are bound to the surface in a static manner, and details of the sub-molecular orbital structure can be resolved with STM. The transition from the static state into the rotating state takes place at 259 *K*. The energy of the spinning state with respect to the lowest energy state having coordinated bonds can be obtained from statistics of the molecule’s switching. The binding energy of the molecule in the spinning state can be easily altered by changing the polarity of the bias voltage applied between the STM tip and the surface. The binding energy is reduced by 80 *meV* when the bias polarity of the sample changes from positive to negative with respect to the tip. The results are consistent with the Coulomb blockade model: when electrons travel from the surface to the *C*_60_ molecule and then to the tip, they produce an accumulation of charge on the molecule due to the Coulomb blockade. This increases the electrostatic interaction between the molecule’s charge and a corresponding image charge created on the metallic surface. The energy of this electrostatic interaction is consistent with the energy difference of the spinning state for positive and negative bias voltage polarity.

## Methods

The sample preparation and experimental conditions closely match that of our previous work with *C*_60_ on WO_2_/W(110)^1^. The STM/STS experiments were performed using a commercial instrument from Createc, in an ultra-high-vacuum (UHV) system consisting of an analysis chamber (with a base pressure of 2 · 10^−11^ *mbar*) and a preparation chamber (5 · 10^−11^ *mbar*). An electrochemically-etched tip made out of tungsten single crystal with [100] crystallographic direction along the tip length, was used to record STM images in constant current mode. The voltage *V*_*b*_ corresponds to the sample bias with respect to the tip. No drift corrections have been applied to any of the STM images presented in this paper. A W(110) single crystal, prepared at the Institute of Solid State Physics RAS, was used as the substrate. An atomically-clean W(110) surface was prepared by *in situ* annealing at 1900 *K* in an oxygen atmosphere of 1 · 10^−7^ *mbar*, followed by a series of high temperature flashes at 2200 *K*. The sample was heated by electron beam bombardment and its temperature was measured using an optical pyrometer (Ircon UX20P, emissivity 0.35). The condition of the W(110) surface was verified by LEED and STM before oxidation. Once a clean surface was obtained, the sample was oxidised at 1600 K in an oxygen atmosphere of 1 · 10^−6^ *mbar* for 15 min. The quality of the resulting oxide structure was verified by Low Energy Electron Diffraction (LEED) and STM before the deposition of *C*_60_ molecules. *C*_60_ (Aldrich Chemicals) was evaporated in the preparation chamber isolated from the STM chamber at a rate of about 0.2 ML (monolayer) per min from a deposition cell operated at a temperature of approximately 700 *K*. Before evaporation, the *C*_60_ powder was degassed for about 8 hrs to remove water vapour. The total pressure during *C*_60_ deposition was in the 10^−9^ *mbar* range and the substrate was kept at room temperature. After deposition the sample was transferred into the STM and initially cooled down to 78 *K* before the temperature is raised in order to observe and conduct experiments relating to the rotational phase transition of the *C*_60_ molecules.

## Supplementary information

Supplementary Video

## Data Availability

The datasets generated during and/or analysed during the current study are available from the corresponding author on reasonable request.
